# Metrology for MRI: the field you’ve never heard of

**DOI:** 10.1007/s10334-025-01238-2

**Published:** 2025-03-19

**Authors:** Matt G. Hall, Matt Cashmore, Hyo-Min Cho, Bernd Ittermann, Kathryn E. Keenan, Christoph Kolbitsch, Changwoo Lee, Chengwei Li, Asante Ntata, Katie Obee, Zhang Pu, Stephen E. Russek, Karl F. Stupic, Lukas Winter, Luca Zilberti, Michael Steckner

**Affiliations:** 1 National Physical Laboratory, Teddington, UK; 2 Korea Research Institute of Standards and Science, Daejeon, Republic of Korea; 3 Physikalisch-Technische Bundesanstalt, Berlin, Germany; 4 National Institute of Standards and Technology, Boulder, CO USA; 5 National Institute of Measurement, Beijing, People’s Republic of China; 6 Istituto Nazionale Di Ricerca Metrologica, Turin, Italy; 7 MKS Consulting, Beachwood, OH USA

**Keywords:** MRI, Quantitative MRI, Metrology, Reproducibility, Traceability, Uncertainty

## Abstract

Quantitative MRI has been an active area of research for decades and has produced a huge range of approaches with enormous potential for patient benefit. In many cases, however, there are challenges with reproducibility which have hampered clinical translation. Quantitative MRI is a form of measurement and like any other form of measurement it requires a supporting metrological framework to be fully consistent and compatible with the international system of units. This means not just expressing results in terms of seconds, meters, etc., but demonstrating consistency to their internationally recognized definitions. Such a framework for MRI is not yet complete, but a considerable amount of work has been done internationally towards building one. This article describes the current state of the art for MRI metrology, including a detailed description of metrological principles and how they are relevant to fully quantitative MRI. It also undertakes a gap analysis of where we are versus where we need to be to support reproducibility in MRI. It focusses particularly on the role and activities of national measurement institutes across the globe, illustrating the genuinely international and collaborative nature of the field.

## Introduction

Quantitative methods for magnetic resonance imaging (MRI) have been in existence since the technology’s invention [1]. In the decades since, enormous strides have been and continue to be made across a range of MR-based imaging methods and post-processing approaches [2]. From beginnings in mapping magnetic relaxivities, MRI is now routinely used to measure quantities as diverse as diffusion [3], flow [4], magnetic susceptibility [5], fat fraction [6], iron content [7], and many others. Quantitative MRI (qMRI) is also used to measure volume, shape, and position [8] of tissue features in a wealth of applications. Whilst these do not require quantitative contrast, they are nonetheless measurements derived from images although we will not define them as quantitative MRI for the purposes of this article, which concentrates on quantitative MRI contrast methods.

Despite this, MRI as used in the clinic is typically not quantitative. The overwhelming majority of MRI procedures performed in hospitals are aimed at generating images to be interpreted visually by human experts to provide information for the diagnostic process. Rather than mapping the value of a particular physical parameter across an organ or region, clinical images typically make use of relative contrast, and spatial inference is subjective and relative to other, nearby tissue features [9]. Quantitative MRI is used in individual diagnostic applications such as characterization of liver iron in haemochromatosis [10], but typical clinical usage is more often semi-quantitative such as when a quantitative method is applied in a non-quantitative way (such as the use of diffusion-weighted MRI to diagnose ischemic stroke [11]), or where imaging outputs themselves are not fully quantitative such as dynamic contrast-enhanced (DCE) imaging [12]. Here effective parameters are extracted from images which are not fully reproducible across scanners and sessions.

What is the reason for this apparent disparity? It is certainly not a lack of potential clinical utility. Publications and reviews of qMRI approaches are very commonly motivated by application to pathology, whether it be diagnosis, staging, treatment planning, or assessing impact [13], and whilst clinical applications are not the only possible uses of qMRI approaches (clinical trials, for instance, are also a key area of application), they certainly represent a very major use case. Applications in clinical trials are similarly not widespread, and larger scale research studies such as the UK Biobank [14] mostly employ routine clinical approaches.

One, at least partial, answer to this question is reproducibility. For a quantitative method to be genuinely useful, it needs to be reliable. Blood tests and other chemical assays are widely used precisely because the answers can be trusted—a clinician knows that a blood sample from a patient will be processed to a high standard and that error rates are understood and minimized [15]. This is also true in the delivery of therapeutics—doses either of pharmaceutical agents or of radiation are highly standardized and extremely reliable. Clinicians can trust that the numbers are what they say they are, and strategies calibrated accordingly.

Such reliability and trustworthiness are not an accident or an inherent property of the technologies. This reproducibility is supported by a framework of measurement science which assesses uncertainty, provides consistent calibration, and provides both guidance for practitioners and support for the standards which the field relies upon. This framework is metrology, the science of measurement.

Metrology ensures that wherever you are in the world a meter is a metre, a gram is a gramme, and a second is a Sekunde. Metrology in its modern form has existed for over a century,^1^ it underpins measurements in labs, hospitals, factories, and courtrooms across the world [17] [18, 19], but for various historical reasons metrology is a relatively recent entrant into the field of MRI. This review will describe what metrology is, why it is important, and what work has already been done. It will also take a more forward-looking view on where MRI metrology needs to be to properly support the development of qMRI, its translation into clinical and other applications, and to maintain quality in its day-to-day use.

Metrology is inextricably linked to the development of standards and for this reason we will also include a review of the current standards landscape and cover some new directions. Unhelpfully, the word “standard” has more than one definition in metrological literature (at least in the English language subset of it). Depending on context, it can mean a document describing how or how well a particular procedure should perform or be performed (as in, for example, an International Electrotechnical Commission (IEC) standard [20]). “Standard” also refers to a measurement device or process which is used as a reference to calibrate another measurement device or process [21]. This review discusses both kinds of standards, but for clarity we will refer to the former as “documentary standards” or “written standards” and the latter as “reference standards” or “metrological standards”. Please note that this is not the case in the rest of the metrological literature, however.

The remainder of this document is structured as follows. First, we will describe metrology and how it works, with examples and focus on global metrological structures and mechanisms, and how these are relevant to MRI. We will then briefly review metrology in other fields of medical physics and imaging, before moving on to qMRI and its needs from a metrological perspective. We will then review the current metrological landscape for MRI, including both quantitative applications and more traditional approaches. Finally, we will take a more forward-looking approach and make a case for an improved metrological framework which better supports the qMRI community.

### What is metrology?

Metrology is the science of measurement [22]. Fundamentally metrology is about ensuring consistency and comparability in measurements made in different places, at different times, and by different methods. One of its key contributions is ensuring consistency in units. Through the activities of National Measurement Institutes (NMIs), manufacturers, researchers, practitioners, engineers, the legal profession, and many others can have confidence that no matter where or how a measurement is made Metrology ensures that the Système international d'unités (SI) system of units is consistent and reliable [22]. Key reference texts for metrology are the Guide to the Expression of Uncertainty in Measurement (“the GUM”) [23] and the International Vocabulary of Metrology (“the VIM”) [22] and we will reference these extensively in this work.

Historically, the role of metrology in the practice of MRI has been limited to a few areas such as implant safety testing or the manufacturing process itself [24–26]. MR images as they are used in the clinic are typically not measurements per se. In the research space, however, things are very different. qMRI has been a reality for decades—MRI is capable of measurements of a wide variety of quantities, from relaxivities to diffusivities to susceptibilities, and as noted above even conventional clinical MR images [1] can be used to measure volumes, positions, and shapes of organs or tissue features. In this review we concentrate on the first of these forms of measurement—the formation of parameter maps. This is partly because this is closer to the accepted definition of “quantitative MRI” as it is typically used, and because this is where the bulk of recent metrological development has happened.

Quantitative MRI, of any form, is measurement. Through the application of a specific pulse sequence, readout, and analysis pipeline the quantities derived in either quantitative maps or via spatially extended inference are physical parameters which, with perhaps some exceptions,^2^ can be said to exist independently of the scan process used to measure it. This means that the tools and frameworks of metrology are available to us. Metrology offers the opportunity not just to characterize reproducibility of results, but to understand the limitations of a given dataset.

Metrology’s link to standardisation and documentary standards come from providing evidence of consistency and underpinning reproducibility. Metrology also links to best practice and measurement performance standards which are codified in national and international standards. Documentary standards are extremely important, not least because they are the mechanism by which the manufacturers of commercial products demonstrate regulatory compliance. Standards need to reflect the best-available knowledge of a technique and enable innovation in a particular technology. Standards also require monitoring to ensure that they effectively support new and emerging techniques without hindering the use of existing ones. In the case of measurements, this necessitates that the existing metrological infrastructure is in place to support quantitative measurements, without which there can be no appropriate basis on which to perform any validation.

A key feature of metrology is that it allows the quantitative comparison of measurements of the same quantity made in different ways. This is crucial for assessing the performance of a measurement process, since it allows measurements from the new process to be compared to other existing ones we already have confidence in. One measurement process can be used to calibrate another, despite both being imperfect to one extent or another. We might, for example, calibrate a practical, on-the-ground measurement approach to another which is more precise but less convenient or flexible. This is known as reference metrology, and leads to the idea of a metrological standard—physical objects or processes which have properties characterized by an independent reference process [22]. The process by which we relate the on-the-ground, day-to-day measurement to the most precise method we have available for the realization of the particular unit is called traceability, and this is discussed in detail later in this review.

Reference standards can in principle themselves be calibrated to other, even more precise standards, leading eventually to the definition of SI units. This hierarchical approach allows very large numbers of measurement devices to be calibrated to common standards internationally. This system also avoids the need for a hypothetical perfect ground truth whilst still allowing consistency, something we will describe in detail later. Providing these references and the link to the SI system is the role of NMIs and we will also describe their role in MRI metrology, including the current state of the art and some current lines of development.

If, as a practitioner of MRI, these concepts seem unfamiliar then you are certainly not alone. Aside from applications in device safety, metrology for MRI is largely unknown outside of a relatively small community, but as interest in quantitative imaging and its translation into clinical use grows, awareness of the need for metrology is growing, as evidenced by recent activity at the meeting of the International Society for Magnetic Resonance in Medicine (ISMRM) [27].

### Why we need it

Consistency of measurement is critical for a number of reasons. The first is to ensure and demonstrate reproducibility and underpin the evidence behind new research claims. It is essential that new data can be compared against existing datasets and that experimental evidence from one lab is directly comparable to similar data from a different lab. Without this, science cannot function. Metrology also supports the spread of best practice in measurement. By quantifying measurement performance, it is possible to standardize what is possible in a particular context, and also to make recommendations on which particular method in practice leads to optimal performance.

In MRI, new techniques are frequently developed by researchers and then translated into clinical use by commercial manufacturers who make approaches practical and economical and guide them through regulatory approval. These are critical steps in achieving patient benefits. Consistency and comparability of measurement are essential in supporting effective translation and use. Any new approach that estimates a physical quantity needs to be as reproducible across devices so that the measured value and any changes can be assessed for significance. This is as true of a cardiac T1 value as it would be of a biochemical assay, a temperature, or the orbital trajectory of a satellite. Reproducibility means that differences between patients can be quantified, and individuals can be stratified into groups. Reproducibility is also essential for calculating effect size in a large-scale study such as a clinical trial.

In each of these cases, it is also necessary to compare measurements directly. This means understanding not just the differences between the measured values but also the significance of those differences. Significance of difference is an inherently statistical question and requires an understanding of the uncertainty in each measurement. In strict metrological terms, the difference between measurements is a statistical statement of significance. Measurements are fully described by distributions [19] and difference or similarity is stated in terms of statistical significance. Two measurements can only be meaningfully compared if the measurement uncertainties are understood and quantified for both. Essentially, we are asking if two measured values are likely to have drawn from the same distribution. No two measured values will ever be exactly the same, but by evaluating the uncertainties associated with them, we gain information about the probability distribution a measurement was sampled from and thereby ask how significant the difference is.

Measurement uncertainty is also important when comparing a measured value to a known threshold e.g., a known safe limit for dosage or exposure. A quantified uncertainty provides important contextual information. A measured value may well be below a defined threshold, but if the range of values defined by the uncertainty breaches it, then additional steps should be taken.

Metrology treats measurement performance in terms of uncertainty and bias [28]. These are statistically and quantitatively well-defined terms which allow a fully quantitative approach to measurement performance evaluation. Metrological terminology also tends to avoid using the word “error” (except in certain, well-defined contexts [22]) and the word “accuracy”. The reason for this is that the concepts of both accuracy and error^3^ involve comparing a measured value to an underlying “ground truth”, which is known with infinite precision. Since no measurement procedure can ever produce a value known with infinite precision and would require knowledge of every effect capable of affecting the measurement, a reference ground truth of this kind can never be known and as a result neither the accuracy nor the error of a measurement can ever be fully quantified. However, the uncertainty and the observed bias from a reference value can both be quantified. Rather, metrology seeks to establish a description of a measurement phenomenon where we present a numerical result along with a statistical description of the range of values in which we believe the “true” result resides. Uncertainty is defined as the breadth of the distribution describing a set of repeated measurements of the same quantity. It can be thought of as the limit of information we have about the underlying value. Bias is defined as a systematic shift away from a reference value. Systematic bias is often easier to deal with than uncertainty, since a systematic shift can often be calibrated away once the measurement process is well characterized.

With enough information about measurement uncertainty, we can formulate the comparison of two measurements within a formal hypothesis testing framework and obtain statistical information (such as standard deviations, significance tests, etc.) to help guide decisions and interpretation [23]. A formal statistical comparison framework for measurements also allows measurement equipment to be calibrated. By performing measurements using one approach on an object which has already been characterized with low uncertainty using a different approach, we can obtain information not just about the uncertainties associated with the measurement process, but also about any systematic biases it exhibits. As we will see in the next section, calibration is also critical in ensuring consistency of units.

A fair criticism at this point would be that comparing a measurement to a reference value seems to be just pushing the same problem onto the next measurement—if we need a reference value to assess measurement performance, how can we assess the measurement performance of the reference, and so on? The answer is *by focussing on consistency in units*. For example, two measurement processes with quantified uncertainties that are calibrated to the same system of units will produce comparable results. It is, therefore, critical that all measurement processes agree on the definition of the units.

The fact that comparability between measurements can be almost taken for granted in many fields is testament to the metrological frameworks that underpin them. A 10 mm bolt will fit a 10 mm nut, and if it doesn’t, the user immediately knows that there is a flaw in the manufacturing process of one or both, damaging the reputation of the manufacturer. Indeed, in the field of engineering the principle of tolerancing is born out of an understanding of inherent variation, both in the manufacturing process and the measurement processes used to monitor it. This is especially critical where safety is important. A pharmaceutical manufactured with a certain stated dose must be trustworthy for the physician prescribing it or patients could be harmed. Radiation delivered to a patient undergoing radiotherapy is carefully calibrated and directed to maximize the dose to a tumor while minimizing the dose to healthy surrounding tissue. In each case, uncertainties are carefully characterized and tightly controlled, and equipment regularly audited to ensure its performance remains within accepted guidelines [29].

MRI has been used to make measurements in research studies for decades including relaxivities, diffusivities, chemical compositions, flow fields and susceptibilities. The list is long [1]. In each case an MR scanner is used to apply a pulse sequence which sensitizes received signals to a particular physical mechanism, images are formed and processed, models are fitted, and maps of parameters generated.

To fully and quantitatively assess the performance of a qMRI technique (of whatever kind) and to ensure that the results of one study are fully comparable to those of another, we need the same kind of metrological framework as has been developed in manufacturing, pharmaceuticals, or radiation dosimetry, but one that has been specifically designed for MRI. Such a framework would then underpin not just reproducibility in research work, but other ongoing standardisation and reproducibility efforts and thereby enable the widespread use of qMRI in routine healthcare and clinical trials.

### Traceability and the role of national metrology institutes

Units are the scales we use to compare quantities and outputs. A consistent system of units is absolutely critical to the modern world since it enables consistency, interoperability, trade, and ultimately trust in products and services. The need for consistency in units has been recognized since antiquity and led historically to establishing bodies with responsibility for maintaining it. The earliest known well-defined unit was the Royal Egyptian Cubit, which was defined by a block of black granite established during the building of the great Khufu pyramid in approximately 9000 BCE^4^ [30]. This was a standard for length based on the distance between the pharaoh’s elbow and forearm, plus the width of the palm. Other examples from history include the regulation of lengths and weights during Qin dynasty China in 221 BCE [31], the Platinum metre from revolutionary France [16], and reference length standards at the Greenwich observatory in London [32]; all used as references in industry and trade to ensure consistency. Similar needs were experienced in the US after independence, and as early as 1807 George Washington petitioned congress “to fix the standards of weights and measures” to achieve consistency across state boundaries eventually leading to a set of standards established by George R Hassler [30].

The modern era of metrology began with the establishment of the SI in the twentieth century. This established a set of base units (the second, meter, kilogram, kelvin, ampere, mole,^5^ and candela) with internationally agreed-upon definitions and overseen by the Bureau International des Poids et Mesures (BIPM). These definitions are linked to practical applications of measurement via a set of reference standards maintained by national-level bodies called National Metrology Institutes (NMIs). NMIs provide the link between measurements made in practical, on the ground activities and the internationally accepted definitions of units.

Measurements require units.^6^ For measurements to be fully consistent and comparable, units need to be consistent. The kelvin (or celsius) degree as measured on a thermometer should be consistent with the definition of the SI kelvin. Without a common definition, kelvin or celsius temperatures used in one place, time, or study will be inconsistent with those in another. This leads to the concept of traceability. Traceability means that any given measurement device is connected, via a suitable, unbroken chain of calibrations, to the internationally accepted definition of the relevant SI unit or units. A measuring device which is not traceable to an SI unit is of limited utility.

The SI system defines the seven base units. Since 2019, these have been defined by fixing the numerical values of fundamental constants (such as Plank’s constant, the speed of light, the frequency of a well-known transition in the electronic structure of Cesium, and a few others) to internationally agreed-upon values [33]. These definitions are extremely robust but not necessarily practical for wide deployment, so the BIPM also defines “embodiments” of units which are a set of experimental procedures to establish their values. From these the multitude of other units in modern life such as density, luminance, magnetic field strength, etc. all originate. These are called Derived Units.

The SI system is connected to other applications of measurement via NMIs. The role of an NMI is two-fold: to develop and maintain what are known as primary standards, which are devices which embody the relevant SI unit for practical purposes, and to offer measurement and calibration services which allow other devices to be calibrated to them, providing traceability. In the early years of modern metrology, NMIs typically did only this, and their activities were limited to the SI units alone. Modern NMIs now typically offer a broad range of metrological standards and services to provide traceability in as wide a range of specialties as possible. SI traceable standards and services exist for radiation dose [34] and gas concentration [35], and many NMIs have activities in mathematical and data-oriented applications such as cryptography [36] and AI-based inference [37]. Many NMIs are also involved with Legal Metrology, which covers how one applies regulatory and statutory mechanisms to the enforcement of metrology and metrological guarantees to the legal profession when needed.

Typically, each industrialized nation will have its own NMI. Examples include the US National Institute of Standards and Technology (NIST), Physikalisch-Technische Bundesanstalt (PTB) in Germany, Istituto Nazionale di Ricerca Metrologica (INRIM) in Italy, China’s National Institute of Measurement (NIM),^7^ the Korea Research Institute of Standards and Science (KRISS), and the UK’s National Physical Laboratory (NPL). This is also the list of NMIs globally who (as of 2025) have teams and activities specifically in MRI. Part of maintaining the consistency of the SI system is performing comparisons between measurement capability and primary standards. It is important to benchmark different capabilities against each other, and to identify and remediate any inconsistencies. These are known as International Intercomparisons and NMIs typically perform them regularly, registering the results with the BIPM. BIPM is not a measurement institute and does not maintain standards. It exists as the highest level of metrological organisation, allowing NMIs to compare capabilities and develop new activities.

**Fig. 1:**
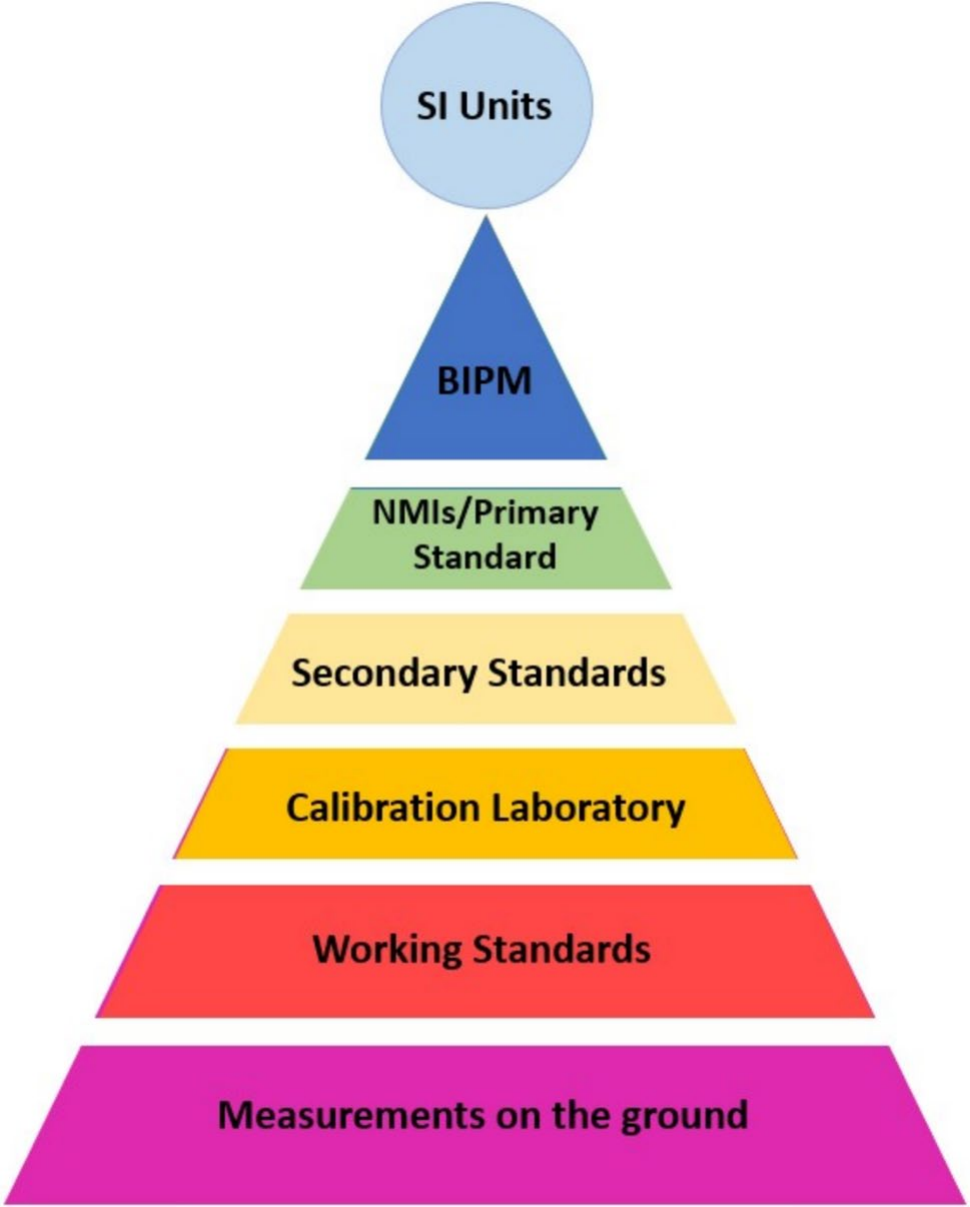
The pyramid of traceability. As we move down the pyramid, the associated measurement techniques have increased uncertainty. Depending on application and quantity of interest, some intermediate steps may be missing

Whilst it is possible for any given measurement device to be calibrated directly to a primary standard, this is typically not how traceability works in practice. A single, national-level institution does not have the capacity to calibrate every measurement device being used. Instead, it is more common for NMIs to calibrate other standards, which are then used to calibrate others and so on. Standards calibrated directly to a primary standard are known (unsurprisingly) as secondary standards, which in turn are used to calibrate tertiary standards and so on. Depending on the scale of the measurement infrastructure, secondary standards may be used by calibration labs, who then offer calibration services to organizations performing measurements in practical applications, often calibrated via working standards maintained in-house. This is referred to as the traceability pyramid, as can be seen in Fig. 1. Each layer of calibration increases capacity in the system since each reference standard can be used to perform many calibrations but leads to an increase in measurement uncertainty.

Organizations engaged in calibration for SI traceability are typically accredited to do so by a national-level body (e.g., the UK Accreditation Service (UKAS)) which audits them for compliance with documentary standards such as ISO^8^ 17025 (the international standard for testing and calibration laboratories) [40], again ensuring international consistency, this time in procedure, quality of workflow, and supporting systems.

### Uncertainty quantification

Uncertainty quantification requires understanding the entire measurement pipeline and quantifying the contributions to the overall uncertainty from each step of the process. A simple example of this would be measuring the length of an item. A simple approach using a ruler would require a) knowledge of a reference standard, such as a gauge block of known size, b) understanding the bias introduced to the ruler by changes in temperature, including a measurement of the temperature and its own uncertainty, and c) the limits of precision on the marks on the ruler. Each of these contributes to uncertainty. Stated together these form what is known as an uncertainty budget [22, 23]. This also considers correlations between effects, which are important when the contributing uncertainties are combined to get the overall uncertainty.

Standard approaches exist to assemble an uncertainty budget, evaluate, and combine contributing uncertainties together. In some instances (such as the one above) this can be calculated analytically, but in other, more complicated cases a better approach is to obtain uncertainties numerically via a Monte Carlo calculation, and this is the approach usually taken in practical applications [23].

Evaluating uncertainty can be challenging, and there is more than one approach to it. One option is to take a “line-by-line” approach where each source of uncertainty is identified and quantified and then combined into a single, overall uncertainty for the complete measurement process. This breakdown of contributing factors is the uncertainty budget [22, 23]. This is the approach taken historically in many areas of measurement science, and it is the approach emphasized in most metrological literature since it gives the clearest insight into the measurement process. It also identifies the sources of uncertainty which contribute the most strongly to the overall uncertainty so that these can be analyzed and ameliorated in future updates. Using this approach, it is also possible to see the overall uncertainty converge on a limiting value, which can also inform strategy for improving the measurement performance.

In some fields, however, the line-by-line approach is not practical. A good example is analytical chemistry, where measurement processes can be extremely complicated (including objects such as spectra formed in several different ways and highly non-trivial model-fitting). Additionally, sources of uncertainty can be too difficult or too numerous to characterize individually. Under such circumstances it is possible instead to work with an overall uncertainty on a final measurement rather than break it down into individual contributions. This approach and has proved extremely useful andthere is considerable existing literature on it, including a suite of ISO standards (ISO 5725) which may prove useful in a qMRI context.

Of course, these two approaches are not mutually exclusive. An overall uncertainty can be pursued using a combination of both methods: quantifying individual sources of uncertainty where possible and comparing their contribution to the overall measured uncertainty to give an idea of how much of the uncertainty is being captured in this analysis, which gives a measure of how much of the measurement uncertainty it is possible to quantify in detail.

In this context it is possible to identify two types of uncertainty: type-A and type-B. Type-A uncertainty is one derived from a quantitative statistical analysis of repeated measurements, whereas type-B uncertainty is one derived by other means e.g., a value from literature, a consensus value, or even a best-guess. Ideally it should be possible to break down a single type-A uncertainty for the overall measurement pipeline into individual components and obtain consistent estimates. This is not always possible, however, and has led to the concept of “dark uncertainty”. A more extensive discussion of these concepts can be found in [41].

### Metrology for radiation dosimetry

As an example of metrology in medical physics and healthcare, this section briefly sketches the role of metrology in treatment planning for radiotherapy. This is probably the most well-developed metrological framework in Medical Physics. Radiotherapy involves delivering a highly controlled dose of ionizing radiation to a specific target location in a patient’s body, maximizing the dose to the tumor while minimizing the dose to surrounding, healthy tissue. Both the amount of radiation delivered and the shape and location of the region where it is delivered are very tightly controlled and as-such require very demanding uncertainties and equipment calibration [42].

NMIs are very closely involved in calibrating and maintaining performance in the linear accelerator (linac) hardware used to plan and deliver radiotherapy. Absorbed dose is traceable to the derived unit the grey (J kg^−1^), which is the energy absorbed in a medium, manifesting as the temperature change measured by a primary standard calorimeter. Since the relevant derived unit through the gray is the joule, it ultimately necessitates traceability to the metre, second, and kilogram. NMIs typically maintain a primary standard calorimeter, which is used to calibrate secondary standard instruments such as ionization chambers which, in turn, are distributed to clinical teams to calibrate their dosimeters. Clinical teams are also audited regularly on their treatment planning procedures to establish how successful their planning and treatment delivery processes are in delivering radiation of the specified does to the specified location and not elsewhere. This is performed using traceable, often anthropomorphic, phantoms.

This metrological framework directly underpins the delivery of radiotherapy, and therefore directly supports not just patient safety but also treatment outcome. The application of metrology gives patients and clinicians confidence that the data used to formulate treatment plans is reliable, and that the dose as delivered will not be biased from what is specified.

### Quantitative MRI and why it is different

Metrology for radiation dosimetry is motived by safety and standard of care and as such is embedded in legal responsibilities of healthcare providers. The situation in MRI is quite different. In this section we will give an overview of the motivation for improved metrology for MRI and some of the potential benefits.

In standard clinical MR imaging, image intensity data is relative, and it holds little to no information about the numerical value of a physical parameter. qMRI goes further than this and uses the scanner (sometimes with additional tools) to make measurements. An obvious question at this point is how well the scanner is performing these qMRI measurements. In particular, what is the uncertainty associated with parameter estimates, are the estimates biased, and how similar are they to measurements made on another device? Without this information, the measurements cannot be compared, and it would be unrealistic to expect consistency between scanners or approaches. With it, however, they certainly can, even if they are made using different approaches on different hardware. Crucially, measurements are rarely (if ever) consistent as a matter of fact, and the history of metrology has shown that careful analysis and referencing is necessary before reaching the point at which measurements are consistent. MRI-based measurement is no different: qMRI needs a metrological framework that is not necessary for purely qualitative imaging approaches [43].

Any measurement of a quantitative parameter that directly relates to an indicator of normal or pathogenic function is called a biomarker. When an MRI measurand (i.e., a property being measured [22]) is used as a biomarker, it becomes even more important that its performance is understood, and its consistency maximized. Clinically, biomarkers are used to diagnose and track the progress of disease [44], and clinical trials can use biomarkers as surrogate endpoints [45]. Whilst in research there are countless MRI biomarkers in development, there are relatively few found in clinical practice or trials [46]. This was previously true of biomarkers from other fields as well—blood tests and other biochemical approaches went through decades of development and several major scandals before a metrologically robust quality control framework was developed to make them reliable, reproducible, and robust [47]. The level of quality assurance required for a biomarker to be routinely usable is high, and it takes effort and resources to develop [48]. Previous and current efforts such as the Radiological Society of North America’s Quantitative Imaging Biomarker Alliance (RSNA-QIBA), the European Imaging Biomarker Alliance (EIBALL), and the Japan Quantitative Imaging Biomarker Alliance (J-QIBA) have begun work here, and already made significant progress, but there is still a need for additional metrological support.

#### The metrology challenges of MRI

There are three main reasons why developing effective metrology for MRI is challenging. The first is the complexity of MRI-based measurement processes. During an acquisition, an MRI scanner repeatedly plays out a pulse sequence to induce an observable signal and sensitize it to a particular physical process of interest. This can be more or less complicated depending on the contrast mechanism and will typically also contain features such as crusher gradients designed to minimize and eliminate other, undesirable effects that would otherwise contribute to the signal. In addition, an imaging sequence is applied, where spatial relationships are encoded and contributions to the signal targeted on subregions of the image. This leads to a signal received via classical induction in a coil which describes the image in k-space. This is then transformed to image space, further processed using a variety of different techniques, and then used as input to some form of image processing to extract the actual quantities of interest [9]. Different choices can be made at all stages of this pipeline, some within the control and visibility of a typical user and some not, some revealed by a particular manufacturer and some not. This leads to hundreds or even thousands of parameters to be defined and set, but to understand MRI-based measurement, we need to understand how each of these steps of the pipeline performs. This is further complicated by the proprietary nature of much of the software provided alongside hardware installations.

The second challenge for MRI metrology is the number of different types of measurement we can make. The above pipeline is a generic description of MRI acquisition and measurement, but there are many different versions of the pipeline, most of which are not particularly standardized. We need to understand not just one highly complex measurement pipeline, but many [46]. This is highly challenging metrologically: how can we design a framework that supports so many measurands whilst allowing for the range of choices made in typical MR acquisitions and processing? As we will see, this question is at least partially answered, but further development is needed to place things on a sustainable and extendable footing.

The third and arguably the toughest challenge is that the purpose of MRI, modality and hardware alike, is clinical diagnosis. Metrology always comes at a cost, be it time, effort, or money, and at the end of the day it is always physicians and not physicists who must decide upon the cost–benefit ratio of quantitative MRI. Only where the benefit is considered high enough will the budget for MRI metrology be available.

## The metrology we have

### Safety and SAR

Historically, the area in which metrology has contributed the most to MRI is safety. This manifests in three ways: (1) ensuring that the energy delivered to a patient during a scan is kept within acceptable limits, (2) checking the compatibility of implanted medical devices, and (3) exposure to static magnetic fields and gradients of both patients and staff.

There has been considerable work done on these exposures at the European level. Before the publication of the European Directive 2013/35/EU on workers’ exposure to electromagnetic fields [49], attention was raised on the exposure due to the movement of MRI operators through the stray stationary magnetic field of the scanners. INRIM developed dedicated computational tools to estimate the electric field induced in the moving body [50–54] and used these to calculate the safety metrics recommended in the relevant International Commission for Non-ionizing Radiation Protection (ICNIRP) Guidelines [55] for a number of realistic scenarios [56].

Additionally, MRI scanning is accompanied by safety risks for the patient that need to be controlled. First, these risks need to be quantified. The applied radiofrequency (RF) pulses lead to absorption of RF energy in tissue, the so-called specific absorption rate (SAR). SAR levels need to be limited to guarantee safe operation without excessive tissue heating. While generally well-regulated through standards [57], not all scenarios are necessarily covered and many need further investigation.

For example, parallel transmit systems use RF coils with multiple, independent transmit elements to shape the RF field for a dedicated application. Calculating the SAR per patient is challenging and is the subject of ongoing work [58–64]. Novel safety concepts such as sensor-equipped implants allow individual determination of the RF heating threat for the actual patient in the scanner could become a solution and are being investigated [25, 58–63, 65–68]*.* This can be performed in simulations [65, 69], via bench measurements at different frequencies [69], or within the MRI system. The effect produced by gradient and/or RF field in the body of patients bearing implants have also been extensively investigated, especially in the case of bulky orthopedic prostheses [26, 70–83].

Numerical tools for the evaluation of SAR have been developed [84, 85] including experimental validation studies [86–88] and correlation between SAR and temperature increase [89–91]. Simulations have been applied to investigate the use of metamaterials to make implants suitable to be scanned without producing artifacts [92, 93]. An alternate simulation framework was developed by NIM, which included an efficient whole-body individual modelling method with software SEMCAD from Speag [94, 95] and associated SAR evaluation as recommended in existing standards (IEC 60601–2-33:2022 [57] and NEMA^9^ MS 8–2016 [96]). A review of implant safety and related heating effects can be found in [25].

### The standards landscape

This section provides a general outline of how to support qMRI with documentary standards. The goal is to benefit the patient with reliable qMRI methods via a framework that demonstrates the reliability of qMRI methods at all stages. It is hoped that some qMRI methods have now reached the requisite level of developmental maturity and would benefit from a documentary standard. Beyond the legally mandated responsibilities between device vendors and regulators, the ownership for the various components discussed below may be shared across clinical sites, device vendors and regulators. Such details will inevitably evolve as qMRI moves into clinical practice.

Both documentary and reference standards symbiotically support the continued growth and development of qMRI. Documentary standards (e.g., IEC 62464–1 [97] or the IEC 60601 series [98], both of which are discussed later), as used for medical device regulatory purposes, define recognized measurement test methods and can also provide requirements or specifications for acceptance criteria. Such measurements may then be used by medical device vendors to support marketing claims to regulatory bodies (e.g., the Food and Drug Administration (FDA) in the US, notified bodies in the EU, or the National Medical Products Administration (NMPA) in China) for approval. Compliance with documentary standards requires that measurement performance is demonstrated unambiguously, and as such well-characterized and traceable reference standards are key to demonstrating that devices are performing to current standards and meet regulatory expectation. Thus, the use of both types of standards should provide the confidence that qMRI measurements enable medical staff to make the necessary care decisions.

Documentary standards typically cascade from the international level to regional and national implementations. The major international standards-setting body relevant to MRI is the IEC, which maintains a network of committees with different themes, subcommittees, and working groups staffed by subject matter experts. Members of committees are drawn internationally from practitioners, manufacturers, engineers, and regulatory bodies. International committees are typically mirrored at the national and regional levels to allow input on new standards and proposals from as broad a stakeholder group as possible. Other bodies, such as the Institute for Electrical and Electronics Engineers (IEEE) and NEMA are also recognized by regulators in some cases, but for MRI and qMRI the main body is the IEC.

The acceptance of standards at the regional and national levels varies by regulatory jurisdiction. In the EU, for example, directives and regulations like the Medical Device Regulation (MDR) EU 2017/745 define *essential requirements* a product must fulfil before it can be placed on the market. Conformity with these requirements is automatically assumed if the device complies with applicable *harmonized* standards. The term ‘harmonized’ means the respective standard has been checked and approved at the EU level so that their structure and wording are consistent with other EU legislation, regulation, and directives [49, 99]. Other nations and regions work directly with the IEC wordings but also issue national and local directives to tune it to the specific context. This is the case in China, for example, where standards are augmented with additional Chinese-language documentation and guidance [100]. Most countries have a local standards body that mirrors relevant subcommittees on the global level. Some examples of these are the British Standards Institute (BSI), Korean Standards Association (KSA), and the Italian Electrotechnical Commission (CEI). The activities of these can vary, but typically they include local publication of international standards as well as representing national-level issues in the dissemination of international standards. Issuing a new or revised standard requires a positive vote from the IEC member countries.

#### Current standards

The major international standards relevant to MRI are the IEC 60601 series [57], which is a safety standard covering all medical electrical devices, and IEC 62464–1 [97] which provides specific image quality standards for MRI (but not quantitative MRI). The IEC 60601 series is a very broad collection covering a wide range of devices (in principle, any electrical device used in a healthcare setting) and covers safety issues with careful attention paid to the scope of individual devices and which parts of the standard are relevant to them. The series consists of a base standard IEC 60601–1, applying to all devices, 12 *collaterals* (IEC 60601–1-X) about certain specific, but not device specific, safety and performance aspects, and a list of around 80 *particulars* IEC 60601–2-Y, dealing with one specific device type. The particular standard for MRI is IEC 60601–2-33 “*Particular requirements for the basic safety and essential performance of magnetic resonance equipment for medical diagnosis*”. It is of such crucial importance, that it is often simply referred to as *the* IEC standard in the MRI community.

The IEC 60601 series is a broader standard than just MRI and applies to other medical devices as well as MRI. Some sections, such as IEC 60601–2-33 pertain to MRI only [98] and enhance or supersede the general standard. As currently defined, the MRI standards does not specifically cover qMRI approaches, although there is interest in developing coverage where appropriate and once techniques are of sufficient maturity to warrant it. It is also worth noting that the user manual for a typical MRI scanner often states that the scanner is not a measurement device [101], a fact that is often surprising to researchers developing quantitative methods, who are known to feel differently [102]. “Special diagnostic procedures” using MRI are also not addressed in the scope of IEC 62464–1. Standards are also under constant review and undergo periodic redrafting exercises. At the time of writing, IEC 60601–1 is undergoing a substantial revision [51], and revisions to IEC 62464–1 are being contemplated.

A further series of standards from American Society for Testing and Materials (ASTM) International deals with the safety aspects in the context of foreign objects in or near the MR scanner. Such items can be external, e.g. a physiological monitoring device with electrodes attached to the patient’s skin, or internal, like a medical implant. ASTM F2503 was groundbreaking in introducing the terminology which was later adopted in many other standards. Foreign objects are classified as i) *MR unsafe* (“an item that is known to pose hazards in all MRI environments"), *MR conditional* (“an item that has been demonstrated to pose no known hazards in a specified MRI environment with specified conditions of use"), or *MR safe* (“an item that poses no known hazards in all MRI environments"). Importantly, image artifacts are considered (diagnostic) safety hazards which automatically excludes all metallic devices from an *MR safe* label. ASTM F2052 and F2213 define test methods to investigate the magnetic displacement force and torque, respectively, on medical devices in an MRI environment. Such investigations are highly relevant, safety-wise, but technically more or less straightforward and not an active field of metrological research. ASTM F2182, entitled *Measurement of Radio Frequency Induced Heating On or Near Passive Implants During Magnetic Resonance Imaging,* describes standardized test methods and equipment to investigate the thermal response of passive metallic implants to RF exposure from the MRI scanner. These ASTM standards were co-developed by the US FDA and are tailored to provide the information FDA needs during the medical device review process. The procedures described in ASTM F2182 for passive implants are also helpful to perform validation experiments as required by the ISO/TS 10974 standard for assessing the hazard from active implants in an MRI environment, issued by the International Organization for Standardization (ISO).

ISO/TS 10974 provides manufacturers of active implants with defined procedures and methods to assess the MRI compatibility of their devices. In many respects it is the active-implants counterpart of the ASTM series on passive implants. The standard, for some tests, applies a tiered approach with simple assessment procedures but large safety margins in Tier 1, allowing a simple and cheap investigation of implants with little hazard potential, up to the extensive (and expensive) full-scale assessment of Tier 4 as in the case of RF-related evaluations.

Medical device vendors both “verify” and “validate” the device operation. Verification is an engineering concept that confirms the device under test (DUT) meets specified requirements. Validation is a demonstration that the DUT fulfils the requirements of its intended use (e.g., the confirmation that a medical device meets a clinical need). For example, a test object purporting to replicate the diffusion value range of a particular organ has been verified and validated. If such a connection between test object and clinical need does not exist, there is little or no proof that the clinical measurement is reliable in the absence of the test object. Collecting the clinical and test object measurement simultaneously is a possible solution. If the test object measurements do not meet expectation, then the clinical measurements may be suspect.

In addition to the international and national documentary standards, there are also recommendations produced by various professional bodies, such as the American College of Radiology (ACR) and the American Association of Physicists in Medicine (AAPM). Whilst the national- and international-level standards are often entwined in regulatory and legal metrology, it is worth noting that recommendations produced by these other bodies do not confer any legal obligation or responsibility regarding the standard of care recommended, see [103]. The ACR [104] provide for both a QA service and also a guidance document on procedures for the testing, which shall be discussed later. American Association of Physicists in Medicine (AAPM) report 100 [105] was developed as an aid to acceptance testing, and offers guidance on a variety of MR system checks as well as mechanical ones, along with indicative performance thresholds/acceptance criteria. The Institute of Physics and Engineering in Medicine (IPEM) produced an extensive report on a wide range of performance tests in Report 112; “Quality Control and Artefacts in Magnetic Resonance Imaging” [104, 106]. Both this document and the suite of relevant MRI Standards from NEMA provide comprehensive detail on a variety of objective tests suitable for quality control and accreditation. It is worth noting, however, that the field of qMRI is not especially well represented in many of the guidance documents available, likely a consequence of the nascency of the field of metrology in these areas.

#### Potential future development

For qMRI purposes, a documentary standard could take various forms. One option is that it could specify a defined test method, including test objects. This would originate from a committee of experts in the relevant fields finding broad consensus on a methodology representative of the best possible realization of the measurand in question and defining the appropriate route to traceability. Alternatively, a less restrictive but no less impactful standard could take the form of defining the minimum level of fidelity to which a measurement must be made. An example of this may be seen in the context of radiotherapy delivery where the International Atomic Energy Agency (IAEA) published a recommendation on the level of uncertainty that should be reached for dose delivery [107].

It is essential that the qMRI test method measurement provides a clinically relevant value. A test object and method that provide a measurement which meets the output expectation of all experts is useless unless it sufficiently mimics clinical reality. Without this it cannot provide reasonable confidence that patient measurements are, by extension, reliable. If a test object and typical human volunteer travelled to many different MR scanners, it is a requirement that the qMRI measurements from both be reliably correct, and the test object measurement must be a reasonable facsimile of human tissue.

Note that while the documentary standard test methods need to be well-defined, and some methodology implementation variance is tolerated, test objects are typically allowed a large range of design options. This requires a careful itemization of all the test object requirements in the documentary standard, but potentially allows for different implementations (e.g., a small versus large version of the phantom in consideration of the range of receive coil sizes). Documentary standards should not be used to promote business interests by mandating specific test methods and test objects, which may be patented. If there is a patent position on some element of a documentary standard, it must be identified via a declaration in the standard.

Artificial intelligence (AI) methods and applications are rapidly evolving, and the implications for qMRI must be considered. Given a regularly imaged test object, the AI-derived qMRI measures may vary over time and across software updates. It is assumed here that a typical constancy test comprising of traditional image quality (IQ) metrics might not detect changes in qMRI measures where AI methods are involved. While a qMRI constancy test needs to identify such temporal variations, the clinical implications of the variations will be unique to the clinical need. What constitutes an acceptable level of qMRI variation may be a site-specific determination. In this regard, a qMRI constancy test has the benefit of an expected target value.

The basic premise that qMRI outputs will influence clinical decision making and perhaps reduce the scope or reduce the need for image interpretation means that if the numeric output is incorrect, inappropriate clinical decisions are possible, and “essential performance” may be at risk. It may be important that the qMRI outputs are demonstrated to be correct in a fashion similar to proving that a medical device emitting radiation is delivering the intended dose to the intended location. Quality assurance and control (QA/QC) of radiation delivery is an excellent example of a well-defined methodology producing measurements that are traceable to reference standards and presumably confirming the outputs are as intended.

It is also essential that the MR scanner itself be maintained to its expected specifications. Traditional IQ metrics-based constancy tests are a helpful tool. A recently restored quenched magnet may no longer have the identical B0 shim due to subtle shifts in the magnet windings, or an eddy current signature may change because the magnet internal temperature shields are now at a slightly different temperature after a helium refill. A gradient amplifier swap may require a pre-emphasis recalibration. All parameters may be within specification but there could still be operational differences in the context of historical continuity. Such constancy tests are ideally quick and simple.

Mature standards are necessary to demonstrate the compliance of a technique to the best available knowledge of how to perform it. There will always be a need for this capacity, and as the size of the field grows so too does the need for appropriate evolution of international standards. Quantitative MRI has the potential to proliferate significantly into clinical usage, and it is vital to ensure the standards landscape enables the translation of promising diagnostic and therapeutic methods.

### Phantoms, new and old

Phantoms play an important role in evaluating MRI system performance, historically providing a controlled method to assess image quality such as contrast resolution, spatial resolution, signal-to-noise ratio (SNR), and geometric distortion. Phantoms are test objects which can be used to evaluate a particular aspect of the image formation or measurement process by providing a suitable reference.

One of the most commonly used phantoms is the ACR MRI accreditation phantom, which was introduced in 1992 and evaluates geometry distortion, spatial resolution, and slice thickness [108, 109] [104, 110]. The ACR phantom was primarily designed for head imaging coils, but it presents significant limitations when applied to other RF coil types, such as breast and knee imaging coils. The Magphan phantom introduced in 2010 provides enhanced capabilities for evaluating geometric distortion, particularly in neuroimaging [111–113]. However, these phantoms are also constrained by their fixed configurations. It is worth noting that these phantoms are used for evaluation of basic system performance in preparation for traditional qualitative investigations. Many of the tests themselves require subjective procedures [104], where different operators would produce differing results based on their levels of experience, access to support software, etc. Any quantitative measurement of spatial characteristics (e.g., distortion, volume, etc.) would need to have appropriate metrology to characterize any inherent variability in the reproducibility of the testing, as well as traceability in the phantom.

In practice, the level of sophistication of the design and construction of a phantom depends on what it is used for. Phantoms to assess image uniformity by eye may be as simple as bottles of water [114]. Conversely, a phantom used to benchmark the performance of a quantitative imaging process should ideally have traceably verified reference values with accompanying statements of uncertainty [4]. In metrological terms, a traceable phantom is a form of reference standard, providing one or more reference values to assess a scanner’s measurement capability. Fully metrological usage of phantoms in qMRI is comparatively recent, though it is becoming more widespread, and there is also an intermediate class of phantoms which aim to provide reliable reference values without providing full traceability.

System phantoms [115, 116] are multi-purpose quantitative phantoms that contain test media (usually gels or solutions doped with paramagnetic ions) to represent a variety of target measurands such as T1, T2, and ADC. Other phantoms are made bespoke to their intended purpose, often homemade, to support local research efforts, or specifically designed for a certain body region, e.g. Alzheimer’s Disease Neuroimaging Initiative (ADNI) phantom for the brain [111] or the T1-mapping and extracellular volume standardisation (T1MES) phantom for cardiac imaging [117–119]. Unfortunately, there is little capability to independently benchmark the properties of these phantoms, nor is it possible to separate out scanner variation from unexpected imperfections in the behavior of the object.

The increasing complexity of MRI systems, driven by advancements such as wireless, flexible, and adaptive RF coils, has created a need for more versatile phantoms. This has led to the development of modular designs, such as the LEGO-compatible Modular Mapping (MOMA) phantom [120] or the iMet-MRI Phantom [116, 121] offering customizable configurations suited to different anatomical regions and coil types [120]. Modular phantoms present a flexible solution for comprehensive MRI quality assurance and quantitative imaging assessments, effectively overcoming the limitations of traditional fixed phantoms. There is also an emerging class of “virtual phantoms” (e.g., [122]) which work by injecting an RF signal into the receiver coil during a scan.

There are various commercial companies who specialize in the manufacturing of phantoms for quantitative MRI 10 and some of these put their products through rigorous metrological evaluation to ensure the high quality and stability of their test objects. This allows for the expected behavior of these phantoms to be part of the traceability chain and guarantee a level of confidence to the end user.

In addition to physical phantoms, digital reference objects and numerical simulations can be a valuable tool. Digital reference objects are mathematical models which capture the details of the measurement process as well as a model of the underlying physics of signal formation [123]. By considering the measurement process and underlying physics in silico, each aspect of the process can be controlled and varied in a way that is not always possible using physical objects. This is valuable in understanding image quality and also in evaluating uncertainties.

A related concept is the digital twin [124], which is a detailed simulation of a device, for example an entire MRI scanner, which aims to be a digital copy of a specific device and is updated as the device is updated to maintain correspondence. Digital twins can also be used to understand measurement performance and evaluate uncertainty and are particularly useful for identifying where important effects contributing to uncertainty or bias may have been overlooked by comparing the digital twin’s performance with that of the live device.

### Electrical properties of tissue

Electric properties tomography (EPT) is a family of qMRI techniques that aim to obtain an indirect measurement of tissue dielectric properties (such as electrical conductivity and permittivity). Interest in these quantities is motivated by the possibility to exploit them as biomarkers (in many types of cancer, the values of conductivity and permittivity tend to increase with respect to the corresponding healthy tissues). Information about these parameters can be used to optimize therapies based on the application of electromagnetic fields and to perform subject-specific SAR assessments.

Work in this area has been led by INRIM, and includes a comparison of EPT techniques [125], uncertainty analysis [126–131], and development of tissue-mimicking materials [132]. A software library is available for the approach (EPTlib—https://eptlib.github.io/) [133], and a study of the tissue dielectric properties, water content, and T1 [134]. The technique’s repeatability and reproducibility have also been investigated using a saline phantom [135].

### Proton density fat fraction

Proton density fat fraction (PDFF) is a widespread technique in the research literature and is also gaining popularity in clinical applications [136, 137]. The technique aims to objectively measure the overall amount of fat in tissue, a parameter which provides information about several pathologies. PDFF is also amenable to phantoms, since the overall amount of fat can be controlled during the preparation of samples. Whilst several commercial fat fraction phantoms exist, many with highly sophisticated materials designed to mimic the NMR spectra of human fat, as far as the authors are aware, none are SI traceable.

To diagnose fat-related diseases such as metabolic dysfunction-associated fatty liver disease (MAFLD) [138] it is essential to quantitatively measure fat content in tissue. PDFF is considered a non-invasive, quantitative imaging biomarker for fat content estimation and is being utilized not only for diagnosis but also as a primary outcome in clinical trials for MAFLD-related drug development [139]Due to differences in implementation across vendors, ensuring measurement repeatability and reproducibility is critical when acquiring multi-center data. To achieve this, water-fat emulsion phantoms are often created [140, 141], or commercial phantoms [142–144] are used. However, these phantoms are not SI traceable and are manufactured with various additives, leading to a lack of data on the chemical fat fraction, homogeneity, and stability of the materials. Therefore, there is a need for reference materials/phantoms with SI traceable homogeneity and stability.

The only NMI currently offering surfactant-free water-fat emulsions as reference materials for fat fraction imaging is KRISS, who have developed approaches for PDFF as part of their modular phantom approach [145], and chemical metrology for fat fraction imaging using a simple fat–water solution was also developed in the iMet-MRI project [116, 121]. In both cases samples with controlled fat and water content are prepared with calibrated balances and using standard reference materials traceable to the Kilogram.

### Key quantitative MRI capabilities

This section summarizes key metrological capabilities either available directly from national measurement institutes or aimed at underpinning services in the future.

#### KRISS reference materials service

KRISS have developed reference materials for qMRI including SI-traceable solutions suitable for T1 and T2 mapping and a surfactant-free emulsified reference material to improve MRI-PDFF (proton density fat fraction) accuracy for multi-site and multi-vendor evaluations [146]. The materials are contained in a modular, Lego-compatible phantom which can be assembled into many different configurations depending on the application and choice of receiver coil. Full details of the approach can be found in [145], which also characterizes homogeneity and stability of the materials. KRISS offer access to these modular phantoms and guidance on their use as a commercial measurement service and they are currently the only NMI to offer reference materials for PDFF approaches.

#### The NIST MRI biomarker measurement service

**Fig. 2:**
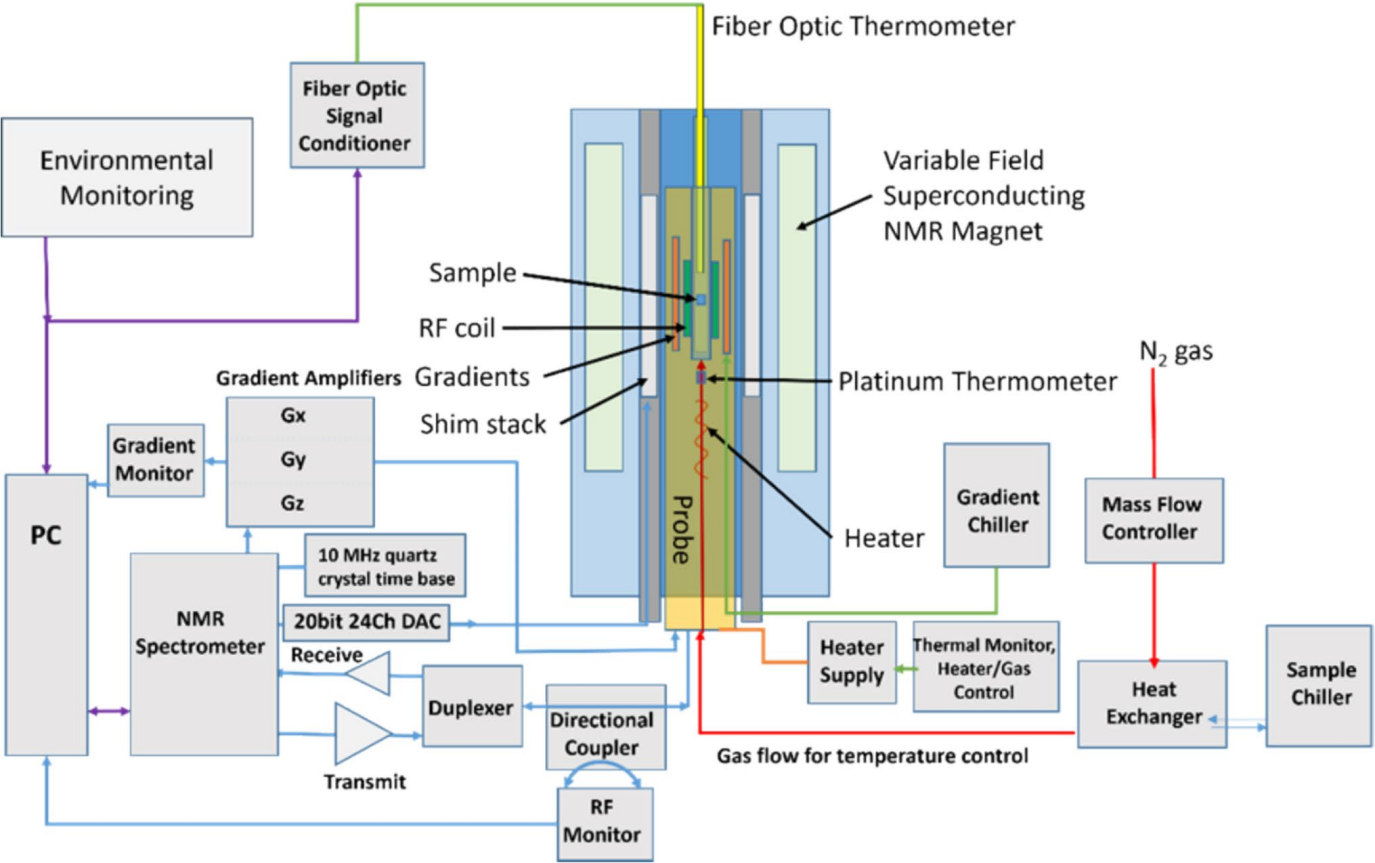
Schematic of the NMR system used for NIST MRI Biomarker Measurement Service

The NIST MRI biomarker service offers traceable measurements of T1, T2, and ADC of material samples at specified field strengths (1.5 T or 3 T) and temperatures. These are provided with fully characterized uncertainties. At present, the measurement service provides calibration of proton relaxation times [147] and isotropic diffusion coefficients [148]. Other biomarkers, where traceability has not yet been established, can be measured as part of a special test where the full measurement protocol is documented and an error is estimated, but a well-defined uncertainty is not provided. For an extensive discussion on the details of the measurement service and traceability, see Ref. [149]. The NIST MRI biomarker measurement service is based on a dedicated metrology NMR system shown in Fig. 2. The system can vary the sample temperature over the range of 0 °C to 60 °C with a 0.2 °C uncertainty. It has 3-axis gradients, a variable magnetic field from 0.5 T to 7.0 T, and a research console (Tecmag Redstone) that allows easy generation and verification of both nuclear magnetic resonance (NMR) and MRI pulse sequences.

For MRI measurements, the chain of calibrations extends back to the definitions of time, length, and temperature. One example of a traceability path is for MRI diffusion measurements, which require a precise measurement of magnetic gradient strength. This is done by taking a MR image of an object with calibrated dimensions, measuring the width in the frequency domain, and then calculating the gradient strength. Here the traceability occurs though calibrating the NMR time base against the NIST cesium atomic fountain clocks and measuring the object dimension using a micrometer calibrated with traceable gauge blocks. Uncertainty is determined using a Monte Carlo Bloch Simulator [149].

#### Phantom libraries

To improve access to phantoms and maximize their usage in the field, both NIST, in partnership with the US National Institute of Biomedical Imaging and Bioengineering, and NPL have phantom lending libraries. These libraries contain commercially available phantoms for multiple MRI measurands including relaxivities, ADC, chemical composition, and more specialist test objects such as diffusion microstructure and kurtosis phantoms. Phantom libraries typically lend items for a fixed period at low or minimal cost, and offer advice on how to use, acquire, and process phantom image data on typical MRI systems.

#### The iMet-MRI project

The development of fully traceable MRI metrology in Europe was largely through the iMet-MRI project. This was a coalition of five European measurement institutes (INRIM, Türkiye Bilimselve Tknolojik Kurumu (TUBITAK), the Institute of Metrology of Bosnia and Herzegovina (IMBiH), and the UK National Measurement Laboratory hosted at the Laboratory of the Government Chemist (NMI at LGC), and led by NPL) which developed traceable materials characterization methods for T1, T2, T2*, ADC, and PDFF including associated chemical metrology for sample preparation and analysis. The project also developed image acquisition protocol and analysis software, demonstrated their performance in an international multi-site trial which included repeated measurements on 1.5 T and 3 T scanners from Siemens, Philips, and GE, and made acquisitions at both 7 T and 0.55 T. Several sets of materials were synthesized, and the results are in the process of being published [116, 121]. iMet-MRI capability is now in the process of being turned into a measurement service facility at NPL using a variable field spectrometer similar to the one used at NIST. An associated metrological intercomparison of the two capabilities is planned once the new facility is complete.

## The metrology we need

Thus far we have concentrated on the current developments of MRI metrology. We have shown that there is an existing body of knowledge and set of expertise globally that provides and underpins measurement capability for MRI safety, and there is also the capability for SI traceability in a small set of MRI measurands via a single primary standard facility at NIST. In this section, we take a more prospective viewpoint and compare current MRI metrology to a fully mature metrological framework, considering the potential benefits and outlining future activities that could take us there.

### Challenges with the current framework

A key issue with current MRI metrology is capacity. The currently employed model for traceability involves commercially manufactured qMRI phantoms containing materials characterized directly on a single primary standard located at NIST. This means that to manufacture traceable phantoms, samples must be sent to NIST for characterization. Since there is an unavoidable variation in batches of materials, this must typically be done on a batch-by-batch basis. In metrological terminology, qMRI phantoms are transfer standards that allow scanner performance to be assessed, but (notably) not calibrate its output.

This arrangement as it stands has the advantage of providing measurements via the smallest possible number of steps between primary standard and measurement-on-the-ground, but suffers from problems of expense, capacity, and fragility. Specifically, primary standard measurement services are expensive, and the necessity of performing batch-by-batch characterization leads to substantial costs for phantom manufacturers which are, of necessity, passed on to customers. This means traceable MRI phantoms are expensive, with little scope for cost reductions through scale or efficiencies in other parts of the manufacturing process. This frequently places phantoms beyond the financial reach of typical clinical imaging departments, even in countries with well-developed healthcare systems, and makes them very much beyond the reach of imaging facilities in less developed nations. This is a significant barrier to the translation and uptake of quantitative MRI approaches and the associated patient benefit.

Having traceability available via a single primary standard globally also leads to risks. First, a single facility, however, efficiently run, means there is only so much capacity for providing measurement services and leads to lengthy waiting times when demand is high. Effectively, it is a cap on growth for the traceable phantom industry. A single facility also means that any technical problems at the facility result in an interruption in availability of services, potentially further lengthening waiting times. This also impacts scheduling of measurement services. If there is a lengthy downtime associated with, for example, a fault or upgrade this would increase the difficulty of scheduling measurements whilst maximizing capacity. All of these considerations exist for any key facility, but the current lack of redundancy in the system makes this risk especially acute.

Capacity issues become even more important if we also consider the need for improved international standards from qMRI. For a new standard to be effective and agreed upon, it must be possible to demonstrate compliance. Since a new standard would (at minimum) specify that measurement performance must be stated and quantitatively benchmarked, traceable measurements would be needed in far greater numbers than are currently available. The same is true for effective translation of quantitative methods into the clinic. Routine QA would be needed to monitor performance, and therefore further increase demand for objects and procedures underpinned by traceable metrology.

Finally, if there is only a single primary standard globally, it is not possible to perform comparisons of performance with other facilities. This means that the ultimate check and balance for measurement performance is not possible, and any drift in measurement performance at the primary standard is difficult to detect.

There is currently considerable support for increased capacity in traceable metrology. NIM and KRISS have developed national-level capability to improve access to traceable references and have expressed interest in increased international co-operation. NPL and INRIM are currently building their own primary standard facilities, aiming to provide similar measurement services to NIST. All of this, while encouraging, will not provide the step-change in metrological framework the field currently requires, however, and there is instead a need for a new traceability structure.

Among regional metrological organizations, Focus Group of Medical Metrology (MMFG) in the Asia–Pacific Metrology Programme (APMP) has expressed willingness to further increase members’ attention to the evaluation and traceability of large medical imaging equipment, including MRI systems. Due to significant differences in economy and healthcare system among APMP members, collaboration with other international or regional organizations has been strongly suggested and welcomed to help improve construction of traceability systems and metrological capabilities among APMP members.

#### Secondary standards

Drawing on the experience of other fields of metrology, one way to achieve increased capacity is to develop secondary standards [28]. As discussed previously, a secondary standard is one that is calibrated directly to the primary, and there has been the suggestion that this could be a good model for MRI metrology [150]. By calibrating a set of secondary standards to existing primary standards, measurement service capacity can be rapidly increased, and costs driven down. Secondary standards for MRI do not currently exist but could be developed with appropriate international collaboration between NMIs and a set of MRI stakeholders (including hospitals, universities, calibration labs, and manufacturers). Furthermore, there is no restriction to a single configuration for secondary standards. Different types of standards suitable for different stakeholders could also be developed.

Secondary standards are typically lower cost than primary facilities, and these could be sited at interested NMIs, existing commercial calibration labs, or at manufacturer facilities themselves. This leverages existing measurement capacity to build the next layer, supporting broader uptake of qMRI methods. In other fields one of the prime advantages to using primary standard calibration, as opposed to entering the chain of traceability further down, is minimization of uncertainties. It may be that this is not as significant an issue for some applications in qMRI, as the measurement uncertainty introduced may be of an order of magnitude lower thanks to inherent patient variability.

#### Accessibility

A very significant challenge for current MRI metrology is access to traceable phantoms by imaging departments and facilities at local hospitals and those in countries with less well-funded healthcare systems. Traceable phantoms are both expensive, and not explicitly required by current standards and guidance [46]. To mitigate this, both NIST and NPL maintain lending libraries whereby phantoms can be borrowed by clinical and research teams. KRISS operate a similar model whereby traceable modular phantoms can be sent to imaging teams to assess scanner performance in quantitative applications, and the phantoms are returned when they are finished [151].

A phantom package in the lending library may consist of far more than just a physical phantom. Ideally the package contains an instruction manual, reference data, recommended imaging and analysis protocols, stability analysis protocols, safety data, recalibration protocols, and a digital twin. The library validates the properties of the phantom, shares data, and curates phantoms. Curation involves monitoring the stability of the phantom properties, fixing or upgrading components as needed, providing verification of both the primary and secondary properties, and maintaining availability for long-term studies.

With recent developments in low field, portable scanning technology such as the Hyperfine Swoop [152] as well as funding from the Gates Foundation, there are efforts to introduce MRI measurement capability into lower and middle income countries. This enables the delivery of improved healthcare in resource constrained areas, such as parts of Malawi [153] where there may not be the requisite infrastructure to support a full scale 1.5 T or 3 T MRI facility. Correspondingly there would not be resources available for high-cost metrology to support any qMRI measurement techniques in these settings, and developing low-cost phantoms and more access to the traceability chain only further spreads the accessibility of potential quantitative techniques.

#### New phantom materials

A very common criticism of current qMRI phantoms is that they are too simple and do not approximate the MR-measurable properties of tissues closely enough. Paradoxically, phantom materials can often lead to measured data which are too close a fit to the assumptions used to analyze them and as a result fail to capture important confounds which are present when applying the same approaches in vivo. A good example of this comes from diffusion imaging, where diffusion in a fluid leads to monoexponential behavior in the measured diffusion attenuation. This is exactly what is assumed by many analysis models (e.g., in calculating an ADC or fitting a diffusion tensor) but is not what is observed in vivo. Here, the interaction between diffusing spins and the tissue environment leads to a slower than exponential signal decay and as such a dependence on measured diffusivities on the overall diffusion sensitization (or *b*-value) [154].

In this case and others like it, the mismatch between the modelling assumptions necessary to obtain parameter values and the actual behavior of the MR signal in vivo is a source of measurement bias, and in many applications we currently lack phantoms sophisticated-enough to evaluate it. The phantoms that do exist are not SI-traceable and many are bespoke items built for a specific research purpose, rather than widely-available products. There is a clear need for metrologically robust SI traceable phantoms which allow these types of effects to be fully characterized.

#### AI in quantitative MRI

In recent years AI has also become the state-of-the-art for MR image reconstruction with methods ranging from image enhancement as a post-processing step to complete image reconstruction using large fully connected networks [130, 155]. Currently the most used techniques combine AI with information about the MR acquisition in a cascaded network architecture [131]. AI approaches have also been presented for qMRI [156–159].

Like any other inference method, it is crucial to understand the uncertainties associated with AI approaches. This is more challenging in some approaches than others, and it is connected to the broader field of AI explainability and trust [160]. In any AI application in medicine, it is critical to understand how uncertain an algorithm is regarding a particular conclusion or value. AI is used in MRI in a number of different ways, from accelerated image acquisition and reconstruction to artefact removal and diagnostic inference [161]. In each case errors made by an algorithm can impact patient outcomes. It is, therefore, critical to incorporate a metrological understanding of algorithmic performance into new frameworks to support the use and deployment of AI.

#### Improved QA procedures

Through the use of available SI traceable phantoms, it is now possible to quantitatively assess measurement performance of individual systems, in particular qMRI applications in T1, T2, and ADC, at least in the phantoms themselves. This immediately raises the question of quality assurance. How should we use this capability to meaningfully assess measurements made in individual studies? There is currently no consensus on this, and although it is not clear currently what form such QA should take and how often it should be performed, it is a topic on which a consensus could be reached. This would require input from a broad range of stakeholders: clinical teams, technologists, clinical scientists, research MR physicists and image analysts, as well as scanner and phantom manufacturers, standards developers, and NMIs.

A closely related question is whether and to what degree it is possible to calibrate measurements acquired in different ways on different systems. In other forms of measurement, it is common to introduce calibration factors to minimize quantified systematic bias [22, 23]. This can have a substantial effect on reproducibility. The extent to which this is possible, feasible, and tractable is an open question in qMRI and will certainly depend on the measurand of interest, but the current metrological framework does make this possible.

It is worth emphasizing that QA is not a one-size-fits-all problem. The level of QA necessary for clinical business as usual will necessarily be quite different than that used in trial qualification and ongoing monitoring, and it will be different again from what an individual research study might require. It is certain, however, that some form of QA for qMRI is necessary. Without QA we cannot meaningfully and quantitatively compare measurements made in different studies and there may be substantial gains from introducing it.

#### Accreditation and trial qualification

Once measurement performance can be fully quantified and benchmarked, it opens new possibilities for scanner assessment and study design. The most immediate possibility is a rigorous quantification of measurement variability for a given device in a particular application, which provides more detailed data about variability per scanner in a trial [112, 162]. The variability in measurements for a particular trial can be more readily evaluated, and as a result there is more data from which to estimate a trial statistical power than would otherwise be available. This cascade enables more confident estimates of the required number of participants for a given effect size. These estimates are currently very challenging for MRI-based assessment and leads to relatively conservative estimates of study size [163]. Quantifying variability in a scanner’s measurement of a particular quantity also means that performance can be monitored and maintained between studies. Additionally, study qualification can be stated in terms of an existing level of performance, reducing the need for lengthy and repeated harmonization phases in larger studies.

Beyond measurement variability for trial qualification, recent work has pointed out that benchmarked scanner performance potentially leads to statistical criteria for measurement performance. The statistical concept of the perfect machine can be brought into an MRI context [150, 164]. The perfect machine is a concept which states that once measurement variability is reduced to a level where it is small compared with the variability of the measurands across a patient cohort, and there is no need to reduce it any further. It should be noted, however, that even when the measurement uncertainty falls below that of natural patient variation, it still may allow for valuable information on long term scanner variation or allow for the detection of emergent faults before they escalate to a point of clinical impact.

When imaging pathology, in many cases the variability of a particular measurand across a patient population is known from non-imaging-based methods and as such enables targets to be set for measurement variability. Hall et al. [150] discuss a grading system for scanner performance in different contexts, and its feasibility goes further and postulates an accreditation scheme for scanners that could be used to recruit sites for clinical trials.

Whilst it remains to be seen whether there is appetite for such a scheme, or how easy it would be to introduce one, this is illustrative of the new possibilities associated with fully benchmarked and traceable measurement performance in MRI.

## Discussion and conclusions

Metrology is a silent partner in many fields of science and engineering. NMIs function as the link between the thousands of measurements that are made every day in modern industrialized countries and a single, unified system of units that allows them to interact, trade, and compare data and results. We have seen that while metrology for MRI is still a work in progress, the principles of traceability and uncertainty quantification are in place for a small number of measurands, and the same principles are in use to ensure device compatibility and patient safety. We have also seen a strong link to the world of documentary standards, where current standards exist for basic image quality, with work underway to develop further unifying standards for qMRI.

There are currently six NMIs globally with an interest in developing MRI metrology, and there is considerable interest from all of them to work together to develop a truly international metrological framework for MRI. In some ways, MRI metrology is at a very exciting stage. Recent work on MRI standardization such as RSNA-QIBA (https://qibawiki.rsna.org/index.php/Main_Page) has highlighted a broad appetite for standardization in many areas of MRI, as have developments in vendor-neutral imaging implementations [165–167]. Improved metrology for MRI has an important role to play in underpinning these and other similar efforts by providing the link to SI units.

The challenge for MRI metrology over the coming years is to transition from the current proof-of-principle phase to a more mature and robust framework, suitable for deployment across many different countries and regions, each with their own priorities and needs. The need for improved standards has been highlighted as critical to unlocking the full potential of qMRI in clinical practice, clinical trials, and to empower AI approaches to imaging and imaging science. To do this, it is imperative that the underpinning metrology has the capacity to support compliance with a new standard. Secondary standards are certainly part of the answer here but so are streamlined approaches to supporting new measurands and studies generating guidance for how to maximize reproducibility.

There are, of course, some questions which arise from metrological approaches to qMRI which cannot be answered by metrologists. One is the question of how equivalent different measurands actually are. For example, are the T1 relaxation times measured by different types of acquisition or fitting model genuinely the same quantity? Questions like these require consensus from the entire community, but it is possible to at least make a suggestion as a point to stimulate debate. In this example we could argue that a T1 as measured by an Inversion Recovery (IR) sequence could be used as a definition of the quantity of interest. We do not always use an IR sequence because it is very time consuming and not always practical and so alternative acquisitions have been developed that attempt to measure the same quantity in a different way, which introduces additional uncertainty and bias. From this viewpoint, alternative acquisition schemes and modelling approaches are approximations of an IR sequence and thus need separate characterization and analysis but nonetheless attempt to measure the same parameter.

An analogy might be drawn here with simpler, more well-established measurement procedures. For example, measuring electrical resistance in a wire or other component. To do this, a current must be applied, but the measured value will be affected by the magnitude of the current. A small current will cause less heating than a large one and lead to a different estimation of resistance, but the actual underlying parameter being measured is the same. The system is perturbed differently by the two different approaches and this needs to be understood, but the measurements are related and comparable. This is discussed in the VIM, Sects. 2.10 and 2.11 [22]. Of course, this is just a starting position for debate and readers may disagree (perhaps strongly).

Characterizing the performance of a given choice of acquisition, modelling strategy, etc. ideally should be part of an individual quantitative study, and in some cases, this may be challenging. Performance in phantoms is certainly part of this assessment, as is potentially the use of digital reference objects to test effects which cannot be captured in physical objects. Again, in some cases this also may be very challenging, but in many other fields this is achieved by calibration, where a device in a particular application is rigorously tested and its performance characterized. The information from a single calibration may then be used in many subsequent studies. The complexity of MRI presents a challenge here: it is certainly not possible to calibrate a scanner in and of itself, but it may be possible to perform a calibration in a particular application. The question of to what extent this is possible is, again, one for the entire community. Metrology can only offer a single viewpoint. Asking what amount of the variability observed in multi-site in vivo studies is due to genuine uncertainty and which is due to differing biases could potentially offer a way to reduce it.

QMRI metrology is an effort that touches a large and diverse group of stakeholders and making sure all voices are heard is imperative. Clinicians, physicists, engineers, technologists/radiographers, scanner manufacturers, phantom manufacturers, regulators, clinical research organizations, pharmaceutical firms, metrology institutes, and patient groups all have their own viewpoints that need to be heard and understood as we go forward, but the same is true of previous metrology and previous standardization projects which demonstrated that this is possible.

There is also the question of who should perform the necessary QA and QC to support qMRI, once it is developed and deployed. This, again, is a topic for debate but we can look to other fields for examples. In radiotherapy, for example, QA and QC is carried out both by manufacturers demonstrating essential performance and safety and by clinical scientists and technologists to confirm and monitor performance as the systems is used. In some cases, QA tests can be automated, but this requires the automated tests to maintain calibration. In current clinical MRI, QA is also performed both by manufacturers, sometimes as part of a service contract, and by clinical science teams, and it seems that this is a basis to proceed from. There is certainly a question regarding how much and how frequent QA for qMRI should be. In research it is likely that many studies will require additional QA over and above any typical clinical routine simply because research studies are more varied than clinical day-to-day scanning. In this case it is the researchers themselves who may need to perform the QA, and arguably the results should be reported in research publications, along with validation experiments and uncertainty analysis.

The biggest challenge may be cultural. Much of MRI operates on a non-quantitative basis, and while there are fields with highly rigorous biophysical focus, it is rare to see a discussion of metrology in the context of verification and validation of a new technique or set of measurements, and the publication of measurement uncertainties is far from universal, even in otherwise-quantitative studies [168]. This can sometimes lead to subjective, qualitative concepts being applied in quantitative concepts. For example clinical recommendations for cardiac T1 mapping [169] require establishing scanner-specific reference values. In a fully quantitative context, we should instead be striving for universal reference values from well-defined metrological procedures. Culture is a more difficult thing to change than technical guidance, but it is perhaps best addressed by demonstrating what metrology and rigorously-reported measurements offer. As with the metrological framework itself, if we build it, perhaps they will come.

## Abbreviations

AAPM: American association of physicists in medicine
ACR: American college of radiology
ADC: Apparent diffusion coefficient
ADNI: Alzheimer’s disease neuroimaging initiative
AI: Artificial intelligence
APMP: Asia-Pacific metrology programme
BIPM: Bureau International des Poids et Mesures (the International Bureau of Weights and Measures)
BCE: Before the common era
BSI: British Standards Institute
CEI: Italian Electrotechnical Commission
DCE: Dynamic contrast-enhanced
DL: Deep learning
DUT: Device under test
EIBALL: European Imaging Biomarkers Alliance
EMPT: Electromagnetic tissue properties
EPT: Electrical properties tomography
FDA: Food and Drug Administration (US medical regulator)
GUM: Guide to the expression of uncertainty in measurements
IAEA: International Atomic Energy Agency
IEC: International Electrotechnical Commission
IEEE: The Institute for electrical and electronics engineers
IPEM: Institute of physics and engineering in medicine
IQ: Image quality
IR: Inversion recovery
ISMRM: International Society for Magnetic Resonance in Medicine
INRIM: Istituto Nazionale di Ricerca Metrologica (Italian NMI)
ISO: International organisation for standardization
J_QIBA: Japan Quantitative Imaging Biomarkers Alliance
KRISS: Korea Research Institute of Standards and Science (South Korean NMI)
KSA: Korean Standards Association
MDR: Medical device regulation
MAFLD: Metabolic dysfunction-associated fatty liver disease
MASLD: Metabolic associated steatotic liver disease
MMFG: Focus group of medical metrology
MOMA: Modular mapping
MR: Magnetic resonance
MRI: Magnetic resonance imaging
NEMA: National Electrical Manufacturers Association
NIM: National Institute of Measurement (Chinese NMI)
NIST: National Institute of Standards and Technology (US NMI)
NMI: National Metrology Institute
NMPA: National Medical Products Association (China’s medical regulator)
NPL: National Physical Laboratory (UK NMI)
NMR: Nuclear magnetic resonance
PDFF: Proton density fat fraction
PTB: Physikalisch-Technische Bundesanstalt (German NMI)
QA: Quality assurance
QC: Quality control
qMRI: Quantitative magnetic resonance imaging
QUIERO: Quantitative imaging enables reproducible outcomes
RF: Radiofrequency
RSNA: Radiological society of North America
RSNA-QIBA: RSNA Quantitative Imaging Biomarkers Alliance
SAR: Specific absorption rate
SI: Système International
SNR: Signal to noise ratio
T1MES: T1-mapping and extracellular volume standardisation
UKAS: UK accreditation service
VIM: International Vocabulary of Metrology

## Footnotes

1 Arguably the birth of modern metrology began in France towards the end of the French Revolution. Seeking an end to the proliferation of mismatching units, the Academy of Sciences tasked a committee to define a new fundamental unit of length; the Meridian. This eventually led to the admission of the infamous Platinum Metre, as well as the Platinum Kilogramme, to be deposited within the French National Archives in July 1799 [16].

2 An argument might be made that relaxivities such as T1 and T2 can only be said to exist for samples placed in a strong magnetic field and as such are not meaningful unless the object is in a scanner. We might further argue that these are phenomenological parameters from a coarse-grained description of underlying quantum processes. The counter to this would be that the relaxivities we measure are a consequence of microscopic molecular motion, which is always occurring, and that nuclear spins will exhibit the same behaviour in the same nominal field regardless of its source (or the manufacturer of its source). The fact that the definition of the parameters comes from the Bloch equations rather than a quantum–mechanical description of spin–spin or spin–lattice relaxation is simply a choice of model and therefore, metrologically, just another source of bias and uncertainty.

3 Unlike accuracy and error, precision is a fully quantitative term. Precision refers to the spread of values if a single measurement is repeated many times and as such can in principle be known without reference to a hypothetical perfect ground truth.

4 Before the common era.

5 The mole was a little late to the party. When the original SI was introduced globally in 1960 there were only 6 units, with the mole being a brought into the family at the 14th Conférence générale des poids et mesures (GCPM) in 1961 [33].

6 We could argue that a percentage or a proportion is unitless but still quantitative, and this would not be unreasonable. To assess the performance of an estimate of proportion, however, we would still require unitful estimates of the various constituents, e.g., total volume of substance of interest and total volume of object. Often our references may be unitful even if the outputs are not.

7 Metrology institutions in China form a hierarchical system consisting of three levels from bottom to top, municipal, provincial, and the national institute. Provincial government can approve provincial or municipal institutes to draft calibration specifications which are used for medical devices evaluation locally as needed [38]. The national institute, also known as National Institute of Metrology (NIM), is responsible for drafting most of national calibration specifications used for evaluating medical devices’ performance and traceability nationwide [39]. When national calibration specifications are issued, these supersede regional and municipal.

8 International Standards Organisation.

9 National Electrical Manufacturers Association.

10 Including but not limited to: Diagnomatic, Poland. Gold Standard Phantoms, UK. CaliberMRI, US. Calimetrix, US. Phantom Laboratory, US. Sun Nuclear, US.
